# High-throughput sequencing for species authentication and contamination detection of 63 cell lines

**DOI:** 10.1038/s41598-021-00779-5

**Published:** 2021-11-04

**Authors:** Oliver Lung, Rebecca Candlish, Michelle Nebroski, Peter Kruckiewicz, Cody Buchanan, Mariko Moniwa

**Affiliations:** National Centre for Foreign Animal Disease, Canadian Food Inspection Agency, Winnipeg, MB Canada

**Keywords:** Biological techniques, Cell biology, Computational biology and bioinformatics, Microbiology

## Abstract

Cell lines are widely used in research and for diagnostic tests and are often shared between laboratories. Lack of cell line authentication can result in the use of contaminated or misidentified cell lines, potentially affecting the results from research and diagnostic activities. Cell line authentication and contamination detection based on metagenomic high-throughput sequencing (HTS) was tested on DNA and RNA from 63 cell lines available at the Canadian Food Inspection Agency’s National Centre for Foreign Animal Disease. Through sequence comparison of the cytochrome c oxidase subunit 1 (COX1) gene, the species identity of 53 cell lines was confirmed, and eight cell lines were found to show a greater pairwise nucleotide identity in the COX1 sequence of a different species within the same expected genus. Two cell lines, LFBK-αvβ6 and SCP-HS, were determined to be composed of cells from a different species and genus. Mycoplasma contamination was not detected in any cell lines. However, several expected and unexpected viral sequences were detected, including part of the classical swine fever virus genome in the IB-RS-2 Clone D10 cell line. Metagenomics-based HTS is a useful laboratory QA tool for cell line authentication and contamination detection that should be conducted regularly.

## Introduction

Cell line authentication is an essential part of ensuring the validity of research and diagnostic results. Misidentified or contaminated cell lines can present irreproducible or inaccurate results which may mislead future research^1^. There have been reports where the cell line was misidentified by the source institute, rendering the results of any publication using that cell line questionable. For example, the KB cell line, believed to be oral or squamous cell carcinoma^2^, and the KU7 cell line believed to be derived from bladder cancer cells^3^, were both found to be HeLa cells. Vaughn et al.^2^ found 631 publications published between the years 2000–2014 that mentioned the use of the KB cell line, of which 574 articles were describing it incorrectly. These, and other papers describing misidentified cell lines, have been and may continue to be cited and used in other studies, thus potentially invalidating the research^2^. As an increasing number of cell lines are reported as being contaminated or misidentified, many scientific journals including Nature and PLOS ONE have now put policies in place for the authentication of cell lines used in their publications^1,4^. Two of the main causes of cell line misidentification are cross-contamination between cell lines and mislabeling of tubes or culture flasks^4^. Cross-contamination may occur within cell lines of the same species (intra-species cross-contamination) or between different species (inter-species cross-contamination). Cell lines may also be completely overgrown and replaced by a contaminating cell line^5^. Regularly confirming the identity of cell lines can prevent contamination and mislabeling errors from affecting future research and diagnostic test results.

One of the methods used for cell line identification is short tandem repeat (STR) profiling, which has been widely used for human identification in forensics^1^. As each human cell line has originated from a different individual, STR profiling allows for differentiation between them. For non-human cell lines, using genes such as the mitochondrial gene *cytochrome c oxidase subunit I* (*COX1*) for DNA barcoding may be used to determine the species of origin. Due to frequent third-position base substitutions in this gene there is a high rate of molecular evolution leading to diversification, which can even differentiate between various phylogeographic groups of the same species^6^. The species can then be determined by comparing the DNA barcode profile of a cell line to databases of these sequences (BOLD, http://www.barcodinglife.org, and NCBI, http://www.ncbi.nlm.nih.gov/genbank/barcode)^1^. However, STR/SNP and COX 1-based methods do not provide information on the presence and type of microbial contamination.

Shotgun metagenomic sequencing allows sequencing of a broader spectrum of DNA or RNA in a sample. Thus, it can be used for species identification of cell lines and potentially detect the presence of bacterial, viral, or fungal contamination. If specific STR and SNP loci are amplified prior to sequencing, STR/SNP profiling may be reliably implemented for the confirmation of the specific cell line. In this study, high-throughput sequencing (HTS) was performed on DNA and cDNA extracts from each of 63 cell lines available at the Canadian Food Inspection Agency (CFIA) National Centre of Foreign Animal Disease (NCFAD) to verify the species of origin and presence of microbial contamination.

## Methods

### Cell culture

A total of 63 cell lines available at the NCFAD were seeded from frozen stocks and grown for 48 h at 37 °C and 5% CO_2_, except for *Trichoplusia ni* cells which were grown with shaking at 27 °C for 24 h before collection. Adherent cell lines were dissociated using 0.25% Trypsin-0.1% EDTA, and cells in suspension were spun down at 4 °C for 10 min at a relative centrifugal force of 600, and re-suspended in culture media. An aliquot of cells was stained with 0.2% trypan blue and counted using a Cellometer Auto T4 counter (Nexcelom Bioscience).

### DNA/RNA extraction

DNeasy Blood and Tissue kit (QIAGEN) was used to isolate DNA and RNA from ~ 2.5 × 10^6^ viable cells using the manufacturer’s recommended protocol. The DNA and RNA were eluted into 50–100 μL of AE elution buffer (QIAGEN). Qubit dsDNA Broad Range (BR) and RNA High Sensitivity (HS) kits (Thermo Fisher Scientific) were used to quantify DNA and RNA in the extracts on the DS-11 FX fluorometer (Denovix).

### High-throughput sequencing

Invitrogen ezDNase enzyme (Thermo Fisher Scientific) was used for the digestion of cellular DNA within extracted nucleic acid prior to cDNA synthesis. Superscript IV First-Strand Synthesis module (Thermo Fisher Scientific) was used for the synthesis of the first strand of cDNA using a 1:1 ratio of random hexamers and oligo-dTs and 300 ng of RNA. The NEBNext Ultra II Second-Strand synthesis module (New England Biolabs) was used according to the manufacturer’s protocol to generate the second strand of cDNA. QIAQuick PCR purification kit (QIAGEN) was used to purify the double-stranded cDNA and eluted in EB buffer (QIAGEN) according to the manufacturer’s recommended protocol. The Qubit dsDNA BR kit (Thermo Fisher Scientific) was then used to quantify the cDNA using a DS11 FX fluorometer.

Sequencing was performed separately on the DNA and cDNA samples with the cDNA samples separated into two runs including a test run with a smaller number of samples due to the timing of the availability of the samples (Tables 1 and 2). Library preparation for the DNA samples was performed using Riptide High-Throughput Rapid DNA Library prep kit (iGenomX) and the manufacturer’s protocol was followed with a 1:1 ratio of the low GC and high GC primers. The samples were pooled and loaded at a final concentration of 18 pM with 1% PhiX, and sequencing was performed on an Illumina MiSeq using a V2 flow cell with a 300-cycle (2 × 150 bp) cartridge.

**Table 1:** Cell lines with species identity determined by sequencing that matched institute records or were previously unknown.

Cell line	Species	cDNA run	Total reads	Number of reads mapped to *COX*1	Breadth of coverage of *COX1* (%)	Mean depth of coverage of *COX1*	Total variants	GenBank reference accession
3T6-Swiss Albino	*Mus musculus*	2	715,514	128	99.3	12.3	0	KY018919.1
A549	*Homo sapiens*	2	2,306,896	1374	100.0	141.8	0	MW389273.1
BHK-21	*Mesocricetus auratus*	2	706,654	39	99.2	3.4	0	EU660218.1
CEF	*Gallus gallus*	2	711,596	1882	100.0	238.5	0	MN013407.1
CHCC-OU2	*Gallus gallus*	2	2,327,710	9681	100.0	1,070.4	0	MN013407.1
CHO-K1	*Cricetulus griseus*	2	3,254,926	300	100.0	20.4	0	KX576660.1
COS-1	*Cercopithecus aethiops*	2	1,438,372	141	99.5	11.0	0	MN816163.1
CPAE	*Bos taurus*	1	5,933,398	1676	100.0	146.6	0	MF663794.1
CV-1	*Cercopithecus aethiops*	2	923,170	277	100.0	22.3	0	MN816163.1
DE	*Anas platyrhynchus*	2	840,902	505	100.0	51.8	0	MH744426.1
DF-1	*Gallus gallus*	2	1,145,492	805	100.0	88.6	0	MK163563.1
Efk-1B	*Eptesicus fuscus*	2	1,015,466	311	95.5	25.6	2	MF143474.1
Efk-2F	*Eptesicus fuscus*	2	1,137,244	257	95.5	20.9	2	MF143474.1
Efk-3B	*Eptesicus fuscus*	2	1,051,616	246	95.4	18.8	2	MF143474.1
EL4-IL2	*Mus musculus*	2	2,016,368	735	100.0	86.3	1	KY018919.1
H1299	*Homo sapiens*	2	1,322,326	392	100.0	37.2	0	MW389273.1
HEK-293	*Homo sapiens*	2	2,997,732	845	100.0	95.0	0	X93334.1
IB-RS-2 Clone D10	*Sus scrofa*	2	4,731,602	2846	100.0	282.8	1	MF183225.1
IPAM 3C10	*Sus scrofa*	2	6,759,802	2827	100.0	238.2	0	MH603005.1
IPAM 3C8	*Sus scrofa*	2	1,701,142	229	100.0	17.0	0	MT199606.1
IPAM 3E8	*Sus scrofa*	2	1,214,488	160	100.0	13.6	0	MG250562.1
IPAM 3F6	*Sus scrofa*	2	1,948,740	434	100.0	33.5	0	MG250562.1
L929	*Mus musculus*	2	1,134,052	164	100.0	21.0	0	EU315228.1
LK-W(14)	*Ovis aries*	2	3,834,432	5324	100.0	613.8	0	EF490453.1
LLC-PK1	*Sus scrofa*	2	862,572	423	100.0	48.2	0	AF486866.1
LMH	*Gallus gallus*	2	847,388	524	100.0	56.5	0	MN013407.1
LT	*Ovis aries*	2	1,165,940	452	100.0	38.0	0	EF490453.1
MDBK-HS-1	*Bos taurus*	2	2,071,902	846	100.0	98.2	0	MN714195.1
MDBK-HS-2	*Bos taurus*	2	658,222	103	100.0	10.9	0	MN714195.1
MDCK SIAT1	*Canis familiaris*	2	1,270,062	506	100.0	53.6	0	KM061581.1
MDCK2	*Canis familiaris*	2	1,474,730	2038	100.0	236.5	0	KM061555.1
MDCK-PGOK	*Canis familiaris*	2	1,242,568	153	100.0	18.9	0	KM061581.1
OA3.Ts	*Ovis aries*	2	1,148,750	647	100.0	55.0	0	KU681212.1
OA4K/s1	*Ovis aries*	2	900,506	182	98.5	13.1	0	MT768116.1
P3X63-Ag8-653	*Mus musculus*	2	1,999,368	2896	100.0	305.4	0	AY533105.1
PaKi	*Pteropus alecto*	2	1,002,720	223	99.9	19.7	0	KF726143.1
PK-15 (PCV-)	*Sus scrofa*	1	1,116,214	1123	100.0	98.1	0	KT279758.1
PK-15 (PCV +)	*Sus scrofa*	1	3,916,490	757	100.0	68.2	0	KT279758.1
QT-35	*Coturnix japonica*	2	824,636	1183	100.0	133.8	0	KX712089.1
RK13	*Oryctolagus cuniculus*	2	729,418	320	100.0	34.3	5	MN296708.1
SC-1	*Gallus gallus*	2	1,296,288	1101	100.0	91.0	0	GU261694.1
SIRC	*Oryctolagus cuniculus*	2	614,400	146	100.0	15.2	1	MN296708.1
SK-6	*Sus scofa*	2	867,492	162	100.0	18.0	0	MG250562.1
ST	*Sus scrofa*	1	2,051,240	707	100.0	60.5	0	AF486866.1
TG180	*Mus musculus*	2	4,749,142	1263	100.0	130.8	0	KP260515.1
Tni	*Trichoplusia ni*	2	1,449,444	348	100.0	43.9	0	NC_045936.1
WSL-R-HP	*Sus scrofa*	2	913,228	804	100.0	65.8	0	MF183225.1
ZZR	*Capra aegregrus hircus*	2	988,600	262	100.0	28.0	0	MH229952.1
**Previously undefined cell lines**								
BD41/31	*Sus scrofa*	2	1,020,194	413	100.0	47.5	0	MH603005.1
MARC-145	*Chlorocebus pygerythrus*	2	594,988	222	100.0	14.6	0	MT481926.1
MRC5	*Homo sapiens*	2	858,666	249	100.0	23.7	0	MK059615.1
N418	*Mus musculus*	2	1,180,212	194	100.0	22.2	0	KY018919.1
OUR-1	*Mus musculus*	2	1,615,188	129	100.0	10.1	0	KP260516.1

Threshold for coverage depth for calling breadth and depth of coverage was set to 3, while the minimum variant frequency = 0.75.

**Table 2:** Cell lines in which a different species from institution records was identified.

Cell line	Species	cDNA run	Total reads	Number of reads mapped to *COX*1/CytB	Breadth of coverage of *COX1*/*CytB* (%)	Mean depth of coverage of *COX1*/*CytB*	Total variants	Reference mitogenome accession
LFBK-αVβ6	***Sus scrofa***	2	4,351,208	**880**	**100.0**	**91.0**	**0**	JN601075.1
	*Bos taurus*			**77**	**44.1**	**8.2**	**98**	MN200869.1
SCP-HS	***Bos taurus***	2	511,706	**202**	**100.0**	**23.7**	**0**	MF663794.1
	*Ovis aries*			**85**	**100.0**	**11.1**	**176**	KU681212.1
**Same genus**								
BGMK	***Chlorocebus pygerythrus***	2	896,826	**501**	**100.0**	**50.4**	**6**	EF597501.1
				320	100.0	40.1	5	JX983774.1
	*Chlorocebus aethiops*			**484**	**100.0**	**48.8**	**47**	MN816163.1
				287	100.0	36.8	47	
CGBQ	***Anser cygnoides***	2	2,426,176	**10,021**	**100.0**	**1,179.6**	**0**	MN356388.1
				3363	100.0	536.1	0	MK102803.1
	*Anser anser*			**10,010**	**100.0**	**1,178.4**	**9**	MN122908.1
				3352	99.9	534.7	19	
MA-104	***Chlorocebus pygerythrus***	2	947,256	**165**	**100.0**	**14.6**	**0**	MT481926.1
				127	100.0	13.5	0	
	*Chlorocebus aethiops*			**149**	**100.0**	**13.2**	**55**	MN816163.1
				96	100.0	9.7	49	
PaLu	***Pteropus ornatus***	2	1,030,394	**197**	**100.0**	**17.2**	**39**	NC_046926.1
				97	100.0	10.1	41	
	*Pteropus alecto*			**192**	**100.0**	**16.9**	**49**	KF726143.1
				96	100.0	10.4	37	
PaSPT	***Pteropus ornatus***	2	936,338	**195**	**100.0**	**25.7**	**40**	NC_046926.1
				93	100.0	12.7	38	
	*Pteropus alecto*			**194**	**100.0**	**25.6**	**50**	KF726143.1
				91	100.0	12.4	37	
Vero	***Chlorocebus sabeus***	2	461,114	**311**	**100.0**	**25.1**	**3**	JQ256913.1
				223	100.0	23.3	2	EF597503.1
	*Chlorocebus aethiops*			**285**	**100.0**	**23.4**	**67**	MN816163.1
				161	99.1	17.9	67	
Vero Nectin-4	***Chlorocebus sabeus***	2	633,176	**238**	**100.0**	**28.0**	**3**	JQ256913.1
				294	100.0	40.2	2	EF597503.1
	*Chlorocebus aethiops*			**207**	**100.0**	**26.2**	**65**	MN816163.1
				223	100.0	33.5	70	
Vero-76	***Chlorocebus sabeus***	1	1,284,012	**618**	**100.0**	**50.3**	**3**	EF597503.1
				598	100.0	64.8	2	EF597503.1
	*Chlorocebus aethiops*			**583**	**100.0**	**47.4**	**67**	MN816163.1
				534	100.0	59.5	70	

Species names in bold are the observed species while the non-bolded names are the expected species based on laboratory records. Bold values indicate results to *COX1* reference while non-bolded cells are to *CytB* reference. Variants were only called if the read depth had a minimum coverage of 3×. Threshold for coverage depth for calling breadth and depth of coverage was set to 3, while the minimum variant frequency = 0.75.

Library preparation for the cDNA samples was subsequently performed with the Nextera XT Library Prep kit (Illumina) following the manufacturer’s protocol due to a switch over of Illumina library preparation methods in the laboratory. In the first run, 26 samples were pooled and loaded at a final concentration of 10 pM with 1% PhiX, and sequencing was performed again on the Illumina MiSeq using a V2 flow cell with a 300-cycle (2 × 150 bp) cartridge. In the second run of cDNA samples, 65 samples were pooled at a final concentration of 18 pM with 1% PhiX, and sequencing was performed on a V3 flow cell with a 600-cycle (2 × 300 bp) cartridge.

### Sequence analysis

iGenomX DNA sequencing reads were demultiplexed using the fgbio^7^ software (v.0.7.0; command used: fgbio DemuxFastqs -i R1.fastq.gz R2.fastq.gz -r 8B12M + T 8 M + T-x metadata.csv). To determine the species of the cell line, metagenomic analysis was performed using the nf-villumina^8^ (v2.0.0) Nextflow^9^ workflow on the concatenated DNA and cDNA sequencing data. As part of the nf-villumina workflow, Illumina PhiX Sequencing Control V3 reads were removed using BBDuk^10^, and poor quality reads and adaptors were removed using fastp^11^. Taxonomic classification of the filtered reads was performed with Kraken 2^12^ using an index of NCBI RefSeq sequences for bacteria, archaea, viruses and the GRCh38 human genome (downloaded and built March 22, 2019), and with Centrifuge using an index of NCBI nt sequences (downloaded and built 2020-02-04). Quality filtered reads were assembled into contigs with Megahit^13^, Shovill^14^, and Unicycler^15^, which were queried against the NCBI nt database (downloaded December 04, 2020) using nucleotide BLAST+^16,17^ (v2.11.0) (default parameters except “-evalue 1e−6”) restricting the search to eukaryotic NCBI nt database entries (i.e. belonging to NCBI taxonomic ID (taxid) 2759). The processed reads for each cell line were mapped against the top matching *COX1* sequence identified by BLAST analysis using Snippy (v4.6.0)^18^ as part of the nf-illmap Nextflow workflow (v1.0.0)^19^. The resulting BAM alignment file was loaded into Geneious v.9.1.8^20^ where a threshold for coverage depth was set to a minimum of three, and variants were called using the Find Variations/SNPs tools with default settings except Minimum Coverage = 3 and Minimum Variant Frequency = 0.75. Variants were only called if the read depth had a minimum coverage of 3×. MDBK-HS-1 is from the cell lines available at CFIA NCFAD in Winnipeg, Manitoba, Canada while MDBK-HS-2 came from the CFIA NCAD laboratory in Lethbridge, Alberta, Canada. For cell lines where the observed species from the top BLAST match was not as expected based on laboratory records, the reads were additionally mapped to the *CytB* gene sequence using the same methods as was used for mapping to the *COX1* sequences.

RNA and DNA viruses and bacteria were identified from the cell line DNA and cDNA sequencing data using DAMIAN^21^. As part of DAMIAN analysis, raw sequencing reads were trimmed using Trimmomatic^22^ with default settings and assembled using SPAdes^23^. Contigs were taxonomically classified using nucleotide BLAST+ (v2.11.0) (DAMIAN BLAST+ option “progressive”) and the NCBI nt database (downloaded December 04, 2020). Trimmed reads were mapped to the viral genomes identified by DAMIAN and additional nucleotide BLAST analysis using the nf-illmap workflow. Variants were called in Geneious V.9.1.8^20^ using the method described above.

## Results

### Cell line authentication

Table 1 lists the cell lines for which the expected species identity was confirmed by mapping the combined reads from the cDNA and DNA sequences to the reference *COX1* gene of the top mitochondrial genome BLAST match for each cell line. All observed species in this list matched the species recorded in the institute’s cell line inventory list. This list also includes five archived cell lines that have been documented as “unknown” which did not have a defined species listed.

**Figure 1:**
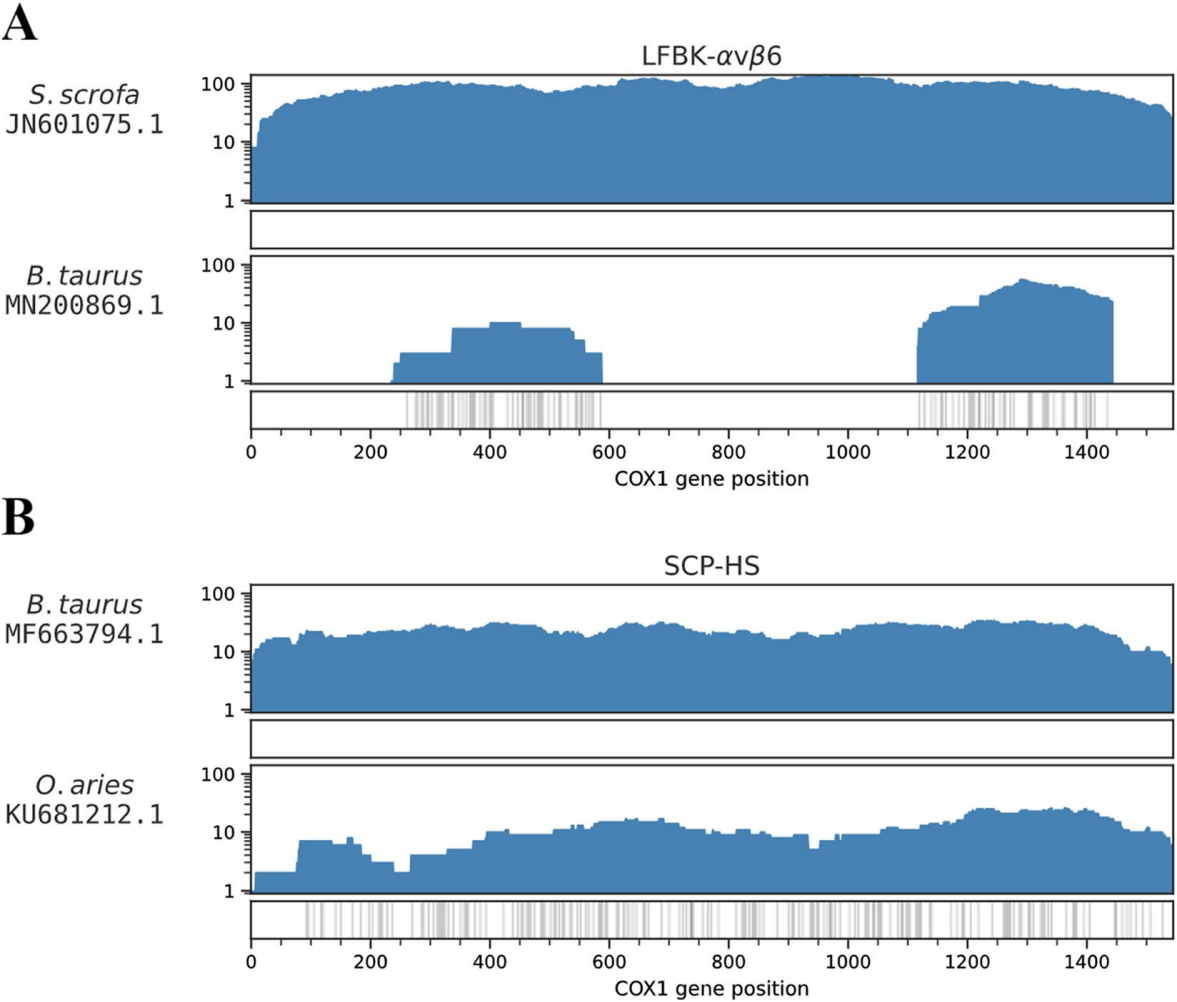
Reference assemblies of LFBK-αvβ6 and SCP-HS reads to references of the expected species and top BLASTn-matched mitogenomes. The nf-illmap workflow was used to map reads from the LFBK-αvβ6 and SCP-HS cell lines to reference *COX1* sequences from the expected species of each cell line and the species which showed the top BLAST match to the de novo-assembled sequences. (**A**) LFBK reads were mapped to *B. taurus* and *S. Scrofa*. (**B**) SCP-HS was mapped to *O. aries* and *B. taurus*. The Y-axis shows the coverage of each genome position. Positions of variants are indicated by the grey lines below the graphs.

Two cell lines, LFBK-αvβ6 and SCP-HS, were determined to be composed of cells from a different species than expected. According to institute documentation, LFBK-αvβ6 was a continuous bovine kidney cell line that constitutively expresses αvβ6 integrin^24,25^; however, there were no BLAST results from the LFBK-αvβ6 de novo assembled contigs that corresponded to the *Bos taurus* genome or mitogenome. All BLAST results matched sequences from the *Sus scrofa* genome and mitogenome. Figure 1A shows the DNA and cDNA reads mapped to reference *B. taurus* and *S. scrofa COX1* sequences. A total of 880 reads from LFBK-αvβ6 mapped to the *S. scrofa COX1* gene and had a breadth of coverage of 100% with 0 total variants (i.e., SNPs, MNPs, and INDELs) between the mapped reads and the reference, while only 77 reads mapped to the *B. taurus* reference *COX1* gene with a breadth of coverage of 44.1% and 98 total variants (see Table 2 for reference accession numbers and results).

According to documentation, SCP-HS is an ovine brain choroid plexus cell line adapted for growth in horse serum; however, the top BLAST results from the de novo assembled contigs were to *B. taurus* and not to *Ovis aries*. Figure 1B shows the coverage of the SCP-HS reads mapped across reference *COX1* sequences from *O. aries* and *B. taurus*. In the *B. taurus* assembly, 202 reads mapped with 100% breadth of coverage across the *COX1* gene with 0 total variants, while in the *O. aries* assembly, 85 reads mapped with 100% breadth of coverage across the *COX1* gene with 176 total variants (see Table 2 for reference accession numbers and results).

Eight cell lines (CGBQ, BGMK, MA-104, PaLu, PaSPT, Vero, Vero Nectin-4, Vero-76) were found to align better to the *COX1* sequence from a different species (within the same genus) than the expected species based on available documentation. For these samples, reads were mapped against the *COX1* sequences from both the expected and observed species. This analysis showed that, when reads were mapped against a reference sequence representing the expected species, more variants were observed than when they were mapped against a reference representing the observed species, suggesting that the cell line is derived from a different species than was expected (Table 2). The *COX1* sequences for the references of the observed and expected species do however share a high similarity; between 95.6 and 96.9% for the primate sequences, 99.4% for the goose sequences, and 97.2% for the bat sequences. A high similarity between the references increases the difficulty in discerning one species from another, therefore for those eight cell lines the reads were also mapped to the mitochondrial gene cytochrome *b* (Cyt*b*) sequence. While the Cyt*b* sequences between the observed and expected species also share a high similarity (between 94.0 and 95.8% for the primate sequences, 98.3% for the goose sequences, and 96.4% for the bat sequences), Table 2 shows that with the exception of PaLu and PaSPT, the results of the Cyt*b* analysis are consistent with those of the *COX1* analysis suggesting with higher confidence that the cell lines are derived from a different species than expected.

### Detection of bacterial and viral sequences

Upon identifying the species of the 63 cell lines, a separate workflow was used to identify bacterial and viral DNA and cDNA sequences. Some viral sequences were expected in the cell lines including human adenovirus C used for the transformation of HEK-293, the common FBS contaminant bovine viral diarrhea virus 2 (BVDV2) in CPAE, and the common porcine circovirus 1 (PCV1) in swine-derived PK-15 (PCV+) cells. Sequences matching these viruses were detected as expected, and PCV1 was also found in all four IPAM clones (Table 3). Retroviral sequences, including murine leukemia virus (MuLV), were also found in some of the cell lines (Table 3). Only viruses with a complete or near complete viral genome (> 98% breadth of coverage) are listed, as incomplete cancer-causing retroviral sequences can be expected within the genomes of tumor-derived cell lines^26^. Reads that were classified as classical swine fever virus (CSFV) were also found in the IB-RS-2 Clone D10 cell line with a 39.5% breadth of coverage across the viral genome with seven total variants relative to the reference genome. In the *T. ni* insect cell line, reads identified as Flock House virus had a breadth of coverage of 85.1% across the reference genome with two total variants (Table 3).

**Table 3:** List of viral genomes detected in the cell lines.

Cell line	Species	Virus	Total reads	Number of reads mapped to virus	Breadth of coverage (%)	Mean depth of coverage	Total variants	Viral reference genome accession
**RNA viruses**								
CPAE	*Bos taurus*	Bovine viral diarrhea virus 2 (BVDV)	5,933,398	173	82.8	2.1	3	MN824468.1
OA3.Ts	*Ovis aries*		1,148,750	27	18.0	0.3	7	MH806437.1
IB-RS-2 Clone D10	*Sus scrofa*	Classical swine fever virus (CSFV)	4,731,602	53	39.5	0.8	7	X96550.1
P3X63-Ag8-653	*Mus musculus*	Murine leukemia virus (MuLV)	1,999,368	3470	99.3	76.3	124	KY574512.1
Tni	*Trichoplusia ni*	Flock house virus	1,449,444	67	87.4	4.5	8	EF690537.1
**DNA viruses**								
A549	*Homo sapiens*	Human adenovirus C	2,306,896	17	3.5	0.1	0	KF429754.1
HEK-293			2,997,732	221	8.8	1.3	1	
PK-15 (PCV+)	*Sus scrofa*	Porcine circovirus 1 (PCV1)	3,916,490	217,311	100	15,571.2	5	MK770354.1
IPAM 3C10			6,759,802	1566	100	80.0	6	MK770354.1
IPAM 3C8			1,701,142	3390	100	186.9	6	MK770354.1
IPAM 3E8			1,214,488	2939	100	174.3	6	MK770354.1
IPAM 3F6			1,948,740	2384	100	143.1	5	AY754015.1

Only retroviruses with > 98% genome coverage were included. Variants were only called if the read depth had a minimum coverage of 3×. Threshold for coverage depth for calling breadth and depth of coverage was set to 3, while the minimum variant frequency = 0.75.

## Discussion

The aim of this study was to authenticate the species identity of cell lines available for use at the Canadian Food Inspection Agency’s National Centre for Foreign Animal Disease, and to establish methods that can be integrated into the laboratory quality assurance system. Confirming cell line species at our laboratory was previously conducted by comparing the electrophoretic migratory patterns of common intracellular enzymes (isoenzymes). Examining the polymorphic isoenzyme profiles between species for cell line confirmation has limitations including limited species range, low sensitivity of detection, and complex data interpretation.

In this study, 53 of the 63 cell lines had a *COX1* sequence that was consistent with the expected species; the reads from each of these cell lines had a breadth of coverage of > 95% across the *COX1* gene, and no more than five variants compared to the reference. LFBK-αvβ6 and SCP-HS cells were found to be from a different genus than expected, suggesting that the cell lines had been misidentified, contaminated, or mislabeled. When reads from the LFBK-αvβ6 and SCP-HS cell lines were mapped to the *COX1* genes corresponding to the species identified by BLAST analysis, no variants were observed in either sample. The porcine DNA found within the LFBK-αvβ6 cell line is consistent with a published erratum that this cell line is of porcine origin^24,25^. LFBK-αvβ6 isoenzyme patterns are also consistent with cultures of porcine origin (unpublished results).

Assembled sequences from eight of the cell lines showed a higher pairwise nucleotide identity to a different species within the same genus than what was expected (Table 2). Five of the cell lines were of primate origin, two were of bat (flying foxes) origin, and one was of goose origin. The number of variants (i.e., SNPs, MNPs, INDELs) between the mapped reads and the *COX1* and Cyt*b* genes were used as an indication of how similar the cell line was to a particular species. The difference in the number of variants between the expected and observed species varied for each cell line (between 9–64 variants for *COX1* and 1–68 for Cyt*B*); however, in each case, the number of variants was higher when aligned to the expected species as compared to the observed species, except for PaLu and PaSPT where the reads mapped to the Cyt*b* gene had a higher number of SNPs to the observed species than the expected. Turner et al*.*^27^ describes the morphological differences between species of the *Chlorocebus* genus of Old World monkeys, and reported that various geographical locations may permit deviation from the predicted morphology of these species. Thus, the species of the individual animal from which each of these cell lines originated was likely misidentified. It was also noticed that the number of variants in the bat cell lines (PaLu and PaSPT) was considerably higher in the observed species (39 and 40 variants, respectively) compared to all of the other cell lines (6 or fewer variants). The genus *Pteropus* is known to be very diverse with a large number of species^28^, therefore, additional investigation will be required to determine if the cell lines are, in fact *P. ornatus,* as identified here, or if there was a misidentification between closely related species when the cell line was originally created.

The current gold standard for the authentication of human cell lines is STR profiling^29^, while non-human cell lines are best identified using DNA barcoding with the *COX1* gene^6^. The International Cell Line Authentication Committee (ICLAC) keeps a Register of all known misidentified or cross-contaminated cell lines. As of this study, the Register was last updated March 25, 2020 and contains a total of 509 cell lines that are misidentified; of these only 38 were nonhuman cell lines^30^. This is likely not because human cell lines are more susceptible to contamination compared to nonhuman cell lines, but rather, because there is more information available for human cell lines in addition to the limitations of STR profiling which is only applicable for single species differentiation^30^. Thus, the method described here is useful since it can identify the species as well as the presence of contaminants such as other cell lines, *mycoplasma,* or viruses^1^.

Experimental results can be negatively impacted due to *mycoplasma* contamination of cell lines. Depending on the species of *mycoplasma*, the effects on the cells vary from changes in protein and nucleic acid synthesis levels to a complete loss of the culture^31^. Detection of contamination is difficult, due in part to the small size (0.3–0.8 µM)^32^ of the *mycoplasma* cells, which allows them to pass through filters^32,33^. Additionally, high concentrations of *mycoplasma* are possible without any obvious visual signs^33^. In this study, *mycoplasma* was not detected in any of the 63 cell lines tested. This result was expected as the NCFAD currently has quality control procedures in place to check for *mycoplasma* contamination in their cultures, and the results here are consistent with the systems in place.

The presence of certain viruses was expected in some of the cell lines. Bovine viral diarrhea virus 2 (BVDV2) is a common contaminant in fetal bovine serum^34^ and was present in the CPAE and OA3.Ts cell lines. Human adenovirus C was found in both HEK-293 and A549 cells. PCV1, a ubiquitous virus in pigs, was found as expected in the PK-15 (PCV +) cell line and in all four of the IPAM clones tested. Retroviral sequences are common in the genomes of their hosts due to insertion into the host genome^25^. The near-complete genome (99.3% breadth of coverage with 77 variants) of murine leukemia virus (MuLV) was detected in the P3X63-Ag8 cell line. Partial genomes from retroviruses such as avian leukosis virus (ALV) and porcine endogenous retrovirus (PERV) were detected in some cell lines.

Sequencing reads covering 39.5% of the CSFV genome were found in the cell line IB-RS-2 Clone D10 with seven total variants shared between the reads mapped and the reference genome. This clone was originally determined to be free of CSFV contamination^28^, however, testing of this cell line obtained from the American Type Culture Collection (ATCC) by Bolin, et al.^35^ detected the virus in this clone. The presence of the entire CSFV genome was also found in the same cell line used by the Pirbright Institute, UK (Don King, personal communication).

## Conclusion

Cell line authentication is important for the reproducibility and accuracy of research and diagnostics involving cell lines as it can help identify unexpected errors and contamination in archived material and cell lines obtained from other sources. This study confirmed the species identity of 63 cell lines that are available at the Canadian Food Inspection Agency’s National Centre for Foreign Animal Disease. Of these cell lines, five were previously undefined, eight were determined to be derived from a different species within the same genus than was expected, and two were identified as species from different genera than expected. The methods described in this study or other comparable methods can be useful as they provide a single approach for species identification, as well as for the detection of contamination (e.g., *mycoplasma*) or the presence of unexpected viruses.
